# Supplementary material for: Seasonal Azithromycin Use in Paediatric Protracted Bacterial Bronchitis Does Not Promote Antimicrobial Resistance but Does Modulate the Nasopharyngeal Microbiome
Source: Int J Mol Sci. 2023 Nov 7;24(22):16053. doi: 10.3390/ijms242216053 (PMC10671346; doi:10.3390/ijms242216053)
Supplement: Supplementary file 1 [file ijms-24-16053-s001.zip › ijms-2688850-supplementary.pdf]

Supplementary Appendix

Table S1. Breakdown of acute courses of antibiotics prescribed for presumed respiratory tract infections during the study

	Number of antibiotic courses prescribed during the study				
	Co-amoxiclav (> 2 weeks duration)	Amoxicillin	Azithromycin	Clarithromycin	Total courses
Azithromycin group	17	14	2	0	33
Comparison group	28	21	3	2	54

Table S2. Number of bacteria species cultured from nasopharyngeal swabs in each group at each swab collection timepoint

Number of bacteria species cultured per swab	Timing of swab in the comparison group (m = months)								
	Baseline	3m	6m	9m	Final 1 12m	Baseline 2 12m	4m⁺	8m⁺	Final 2⁺ 12-20m
0	4	5	1	3	3	2	3	4	7
1	3	3	7	3	5	5	4	3	4
2	3	2	1	4	2	5	2	1	2
3	0	0	1	0	0	3	1	0	1
No swab collected	0	0	0	0	0	0	5	7*	1*

A) Comparison group

Number of bacteria species cultured per swab	Timing of swab in the azithromycin group (m = months)								
	Baseline	3m	6m	9m	Final 1 12m	Baseline 2 12m	4m⁺	8m⁺	Final 2⁺ 12-20m
0	4	6	5	3	4	5	5	5	10
1	4	1	2	2	1	5	1	2	3
2	2	1	2	1	2	5	1	2	1
3	0	0	0	1	0	0	0	0	0
No swab collected	0	1*	1	3**	3**	0	8*	6*	1*

B) Azithromycin group

⁺ Time swab was taken after Baseline 2 swab collected: m - months

*One participant left the study, the remaining did not get a swab collected due to COVID-19 restrictions
** Two participants left the study, the remaining did not get a swab collected due to COVID-19 restrictions.

Figure S1. MIC distributions of M. catarrhalis and S. aureus isolates

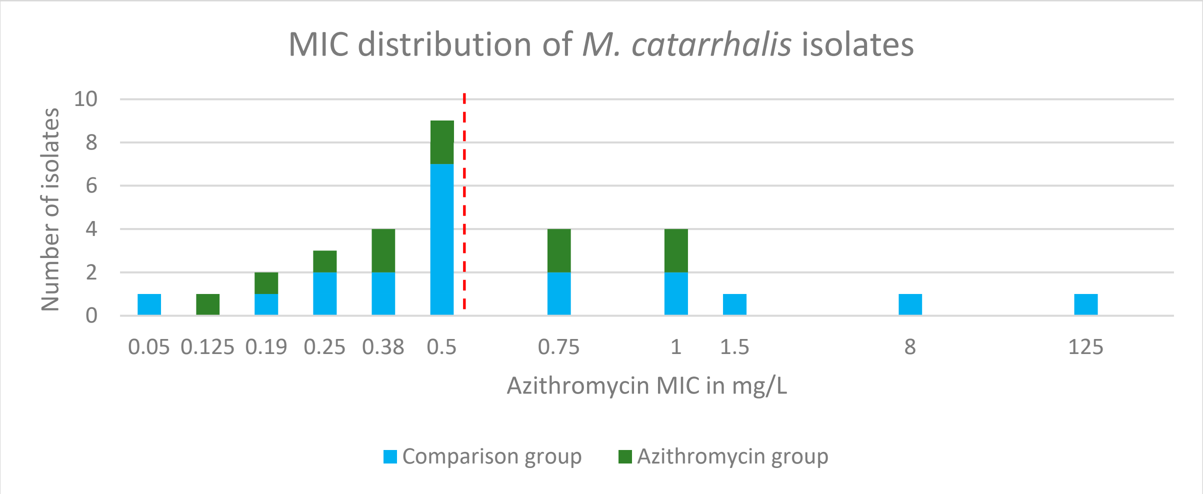

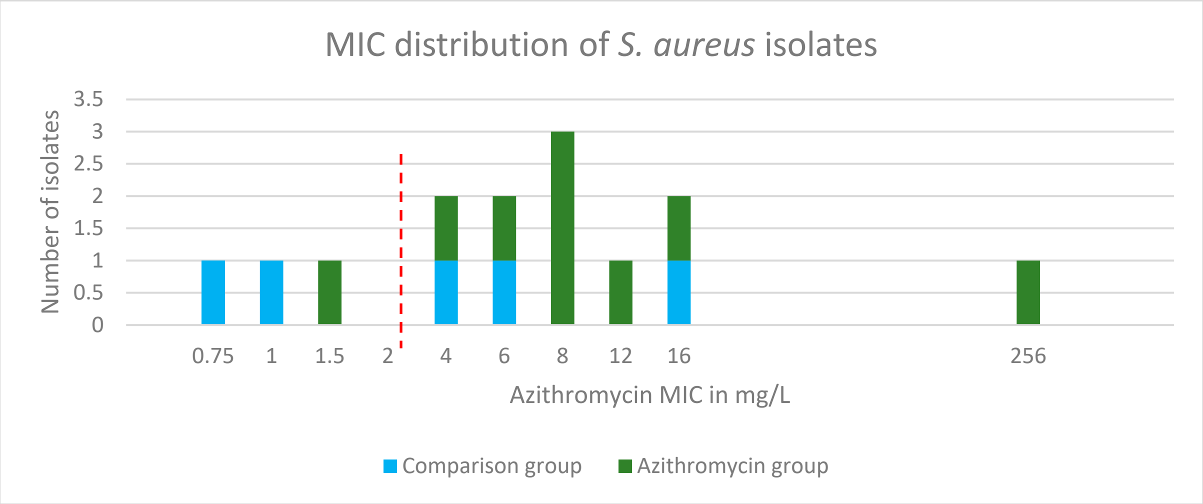

Azithromycin susceptibility was tested using an agar-gradient diffusion method, E-test strips. Susceptibility was determined using the EUCAST break points for each isolate; M. catarrhalis, H. influenzae. Dashed red lines indicate EUCAST breakpoints [27].

Table S3. Repeat parallel susceptibility testing of a subset of *S. pneumoniae* isolates

Sample	*AZM MIC mg/L (first E-test strip)	Erythromycin Disc diffusion in mm (first)	*AZM MIC mg/L (2nd E-test strip)	Erythromycin Disc diffusion in mm (2nd)	Erythromycin MIC Resistant > 0.5mg/L (E-test strip)

1	1	31	1	31	0.25
2	1.5	31	1.5	31	0.19
3	2	29	2	29	0.125
4	0.75	30	0.75	30	0.25
5	2	29	2	29	0.25

EUCAST breakpoints for S. pneumoniae: Azithromycin resistance, MIC > 2mg/L, Erythromycin resistance, MIC >0.5mg/L. Erythromycin resistance, < 19mm

Table S4. Quality assessment of the *S. pneumoniae* genome assemblies provided by 'MicrobesNG'

	Median (25th-75th quartiles)
Number of contigs	29 (24 – 44)
Largest contig	374452 (322588 – 434645)
Total length	2087469 (2051440 – 2119495)
GC(%)	39.57 (39.54 – 39.62)
Mean Coverage (Standard deviation)	80.34 (20.4)

A summary of the quality assurance of the 37 sequenced S. pneumoniae genomes. Descriptive analysis of sequencing showed the mean total length and GC(%) coverage to be consistent with that found in S. pneumoniae genomes.

Figure S2. Sample processing pathway. A flow chart showing the number of samples initially processed and the number removed during the quality control process

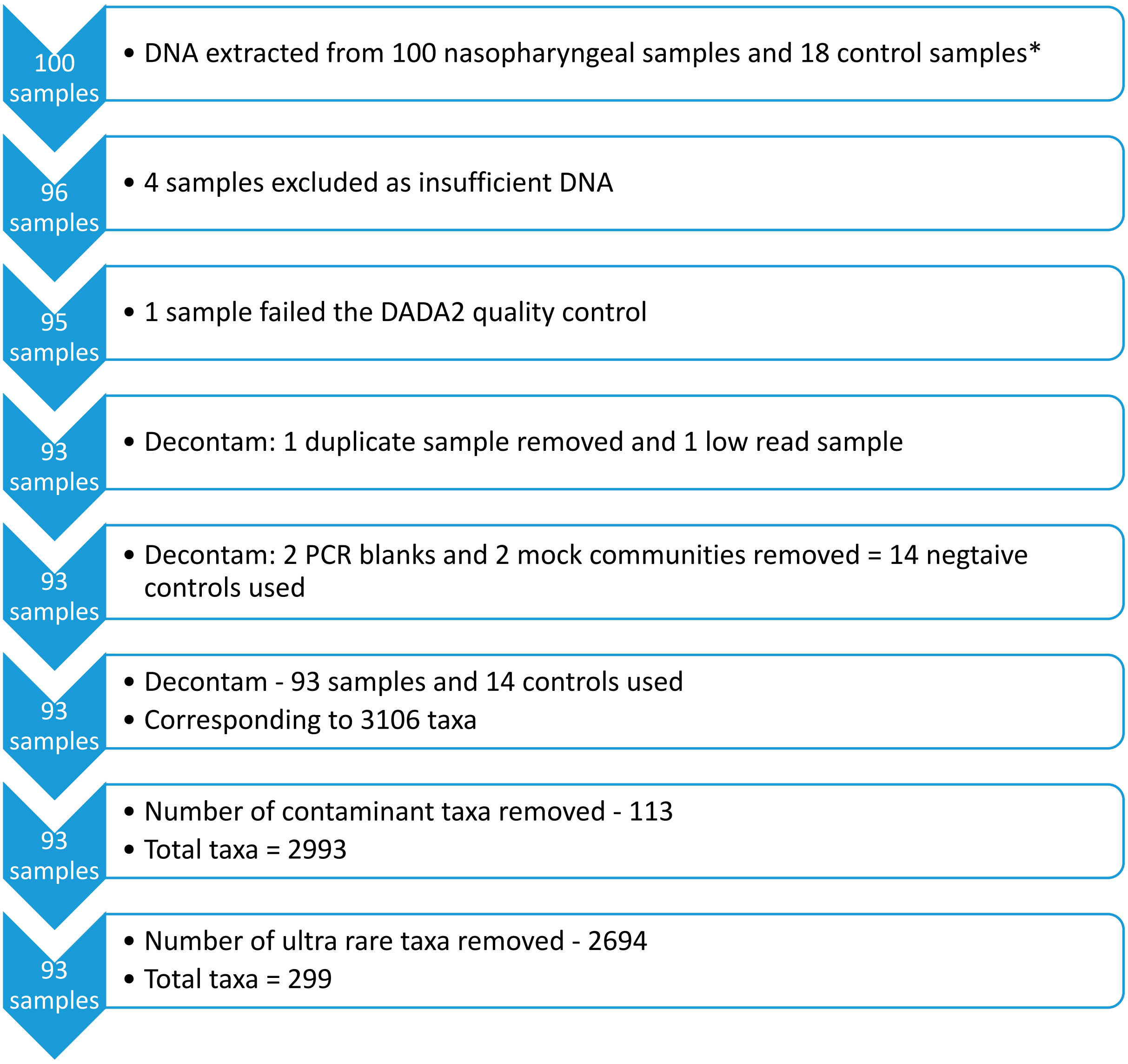

**2 positive mock community samples and 16 negative controls (2 PCR blanks, 4 transport medium samples and 10 ISO_blanks containing reagents)*

Figure S3. Non-metric multi-dimensional scaling of beta diversity for DNPS collected from children in both comparison and azithromycin group

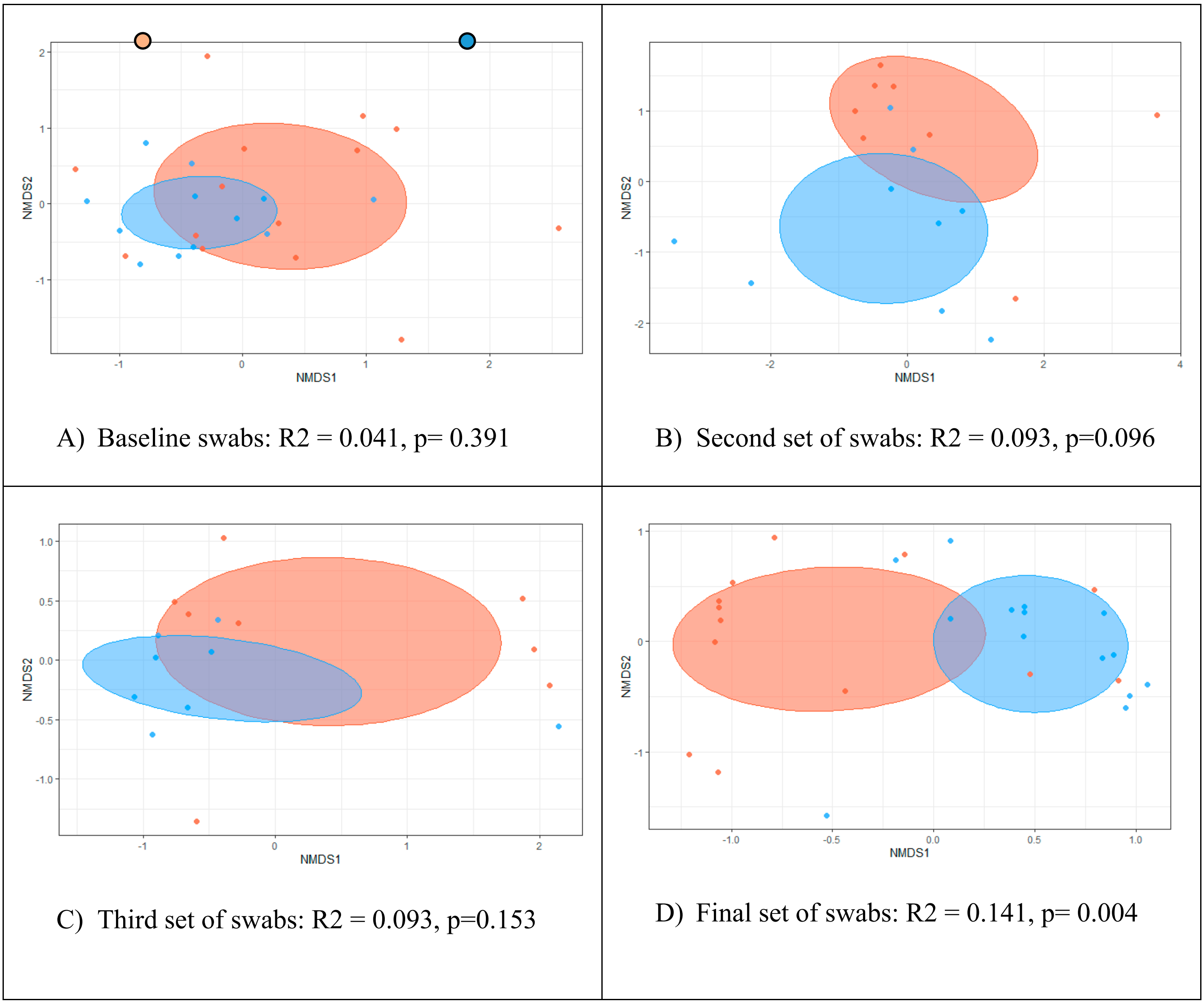

Azithromycin group Comparison group

Beta diversity has been calculated using the Bray-Curtis dissimilarity measure and plotted using Non-Metric Multidimensional Scaling (NMDS). Coloured plots indicate samples taken from participants either in the comparison (blue) or azithromycin group (orange). A-D show swabs taken at different time points over the study period. Significant dissimilarity was only found between the 2 groups in the final set of swabs taken.

Figure S4. Non-metric multi-dimensional scaling of beta diversity: Those actively taking azithromycin at the time of swab collection compared to the comparison group.

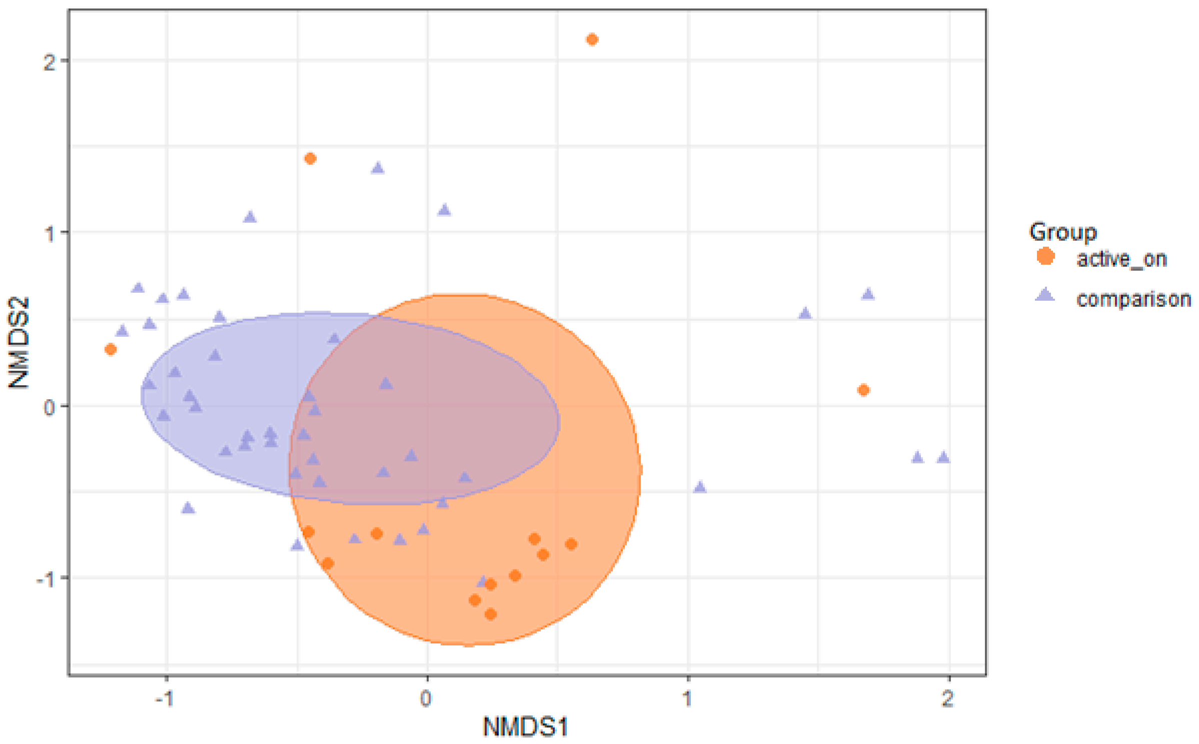

Beta diversity has been calculated using the Bray-Curtis dissimilarity measure and plotted using Non-Metric Multidimensional Scaling (NMDS). Coloured plots indicate samples taken from participants in the azithromycin group, actively taking azithromycin at the time of swab collection, compared to those in the comparison group. Azithromycin treatment had a significant effect on the microbiota composition when those actively taking azithromycin at the time of swab collection were compared only to those individuals in the comparison group. PERMANOVA; R=0.079, p= 0. 001

Figure S5. The microbial composition of nasopharyngeal swabs collected from those in the comparison group

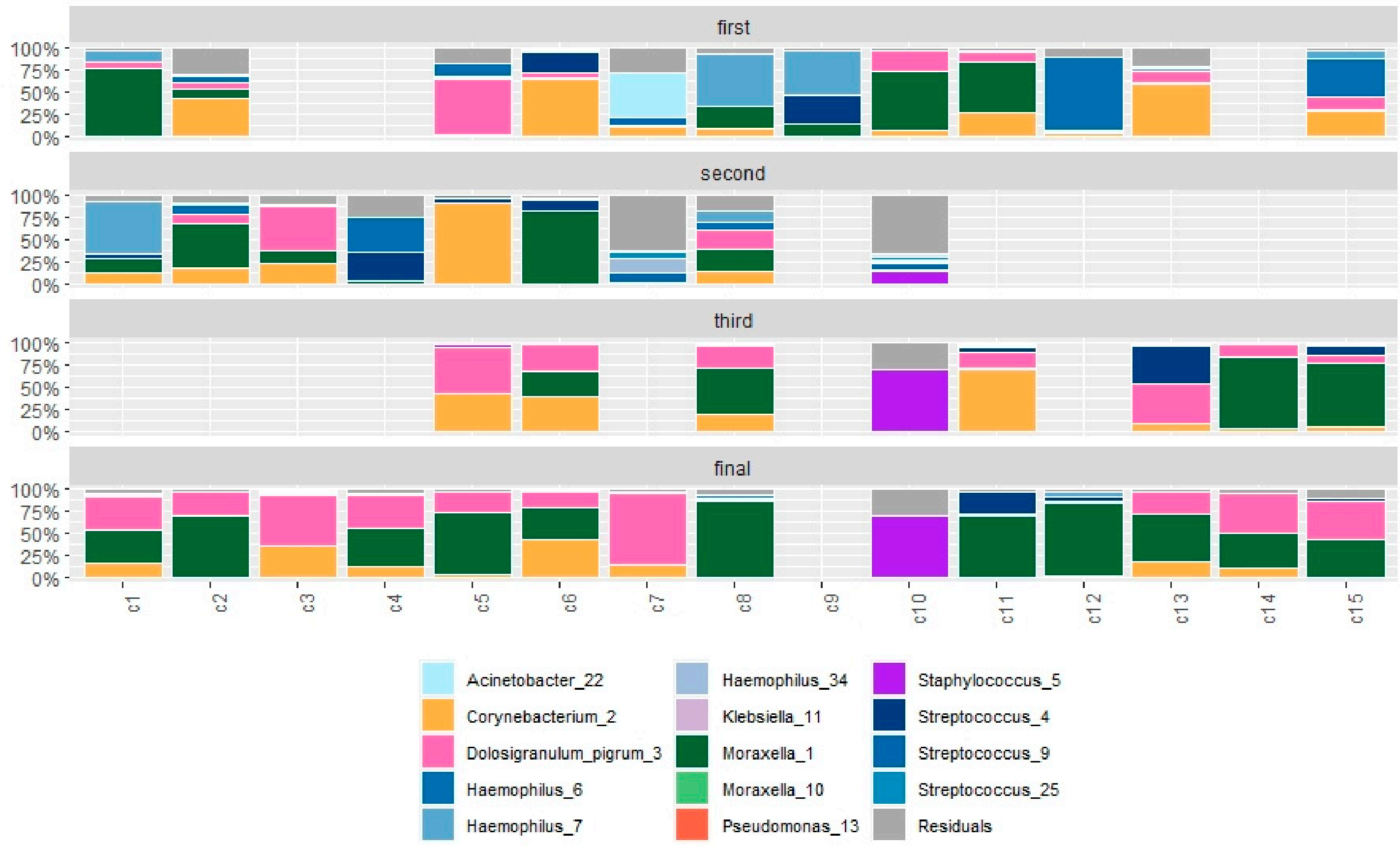

The microbial composition of the top 15 most abundant taxa in the nasopharyngeal swabs collected from children in the comparison group. Baseline swabs are denoted by "first". The 3 subsequent swabs taken over the study period are presented underneath in columns for each participant. Blank cells are present as some swabs were unable to be collected during the COVID-19 pandemic and 3 baseline swabs did not pass the quality control for 16s rRNA analysis. The naming system of the amplicon sequence variants (ASVs) indicates firstly the genus or species if identified. The numerical value following the taxonomy level reports how abundant that particular genus/species was across all the study samples.

A) The microbial composition of nasopharyngeal swabs collected from those in the azithromycin group

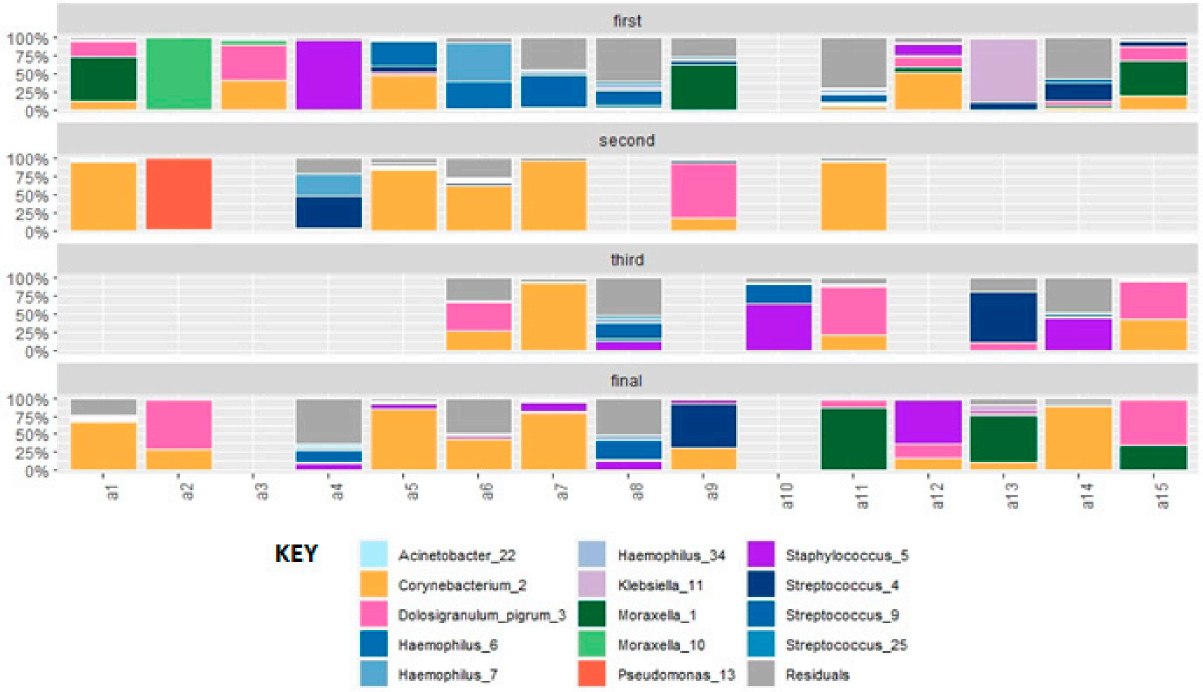

The microbial composition of the top 15 most abundant taxa in the nasopharyngeal swabs collected from children in the azithromycin group. Baseline swabs are denoted by "first". The 3 subsequent swabs taken over the study period are presented underneath in columns for each participant. Blank cells are present as some swabs were unable to be collected during the COVID-19 pandemic and 1 baseline swab that did not pass the quality control for 16s rRNA analysis. The naming system of the amplicon sequence variants (ASVs) indicates firstly the genus or species if identified. The numerical value following the taxonomy level reports how abundant that particular genus/species was across all the study samples.

Table S5. Differential abundance testing between active and comparison groups at baseline, second and final sets of swabs

ASV	Logarithm of fold change	Adjusted p value
Moraxella_lincolnii_19	-8.412139	0.001
Stenotrophomonas_47	-7.294903	<0.001
Moraxella_52	-6.850686	<0.001
Massilia_144	8.523	<0.001
Haemophilus_8	9.105	<0.001
Moraxella_23	11.946	0.001

A) Baseline swab

ASV	Logarithm of fold change	Adjusted p value
Enterococcus_30	-13.078	0.013
Corynebacterium_33	-12.262	0.013
Moraxella_12	-9.794	0.013
Haemopilus_56	-8.603	0.023
Acinetobacter_44	1.354	0.087

B) Swabs collected at 4-months

ASV	Logarithm of fold change	Adjusted p value
Moraxella_1	-5.149	0.001
Dolosigranulum_pigrum_3	-3.594	0.004
Haemophilus_7	-2.839	0.001
Actinobacillus_141	7.227	0.001
Corynebacterium_21	7.463	0.005
Porphyromonas_121	9.407	<0.001
Corynebacterium_18	12.639	<0.001

C) Final set of swabs

Key

Lower abundance taxa in the azithromycin group compared to the comparison group

Higher abundance taxa in the azithromycin group compared to the comparison group

Table S6. Antibiotics used for susceptibility testing using a disc diffusion method for specified bacteria. European committee on Antimicrobial Susceptibility Testing (EUCAST) zone diameter breakpoints used.

Bacteria	Antibiotic (µg on disc)	Zone diameter break points (mm) [27]	
		Sensitive ≥	Resistant <

S. pneumoniae	Erythromycin 15<br>Oxacillin 1<br>Tetracycline 30<br>Chloramphenicol 30<br>Levofloxacin 5	22<br>20<br>25<br>21<br>50	19<br>20<br>22<br>21<br>16
H. influenzae	Chloramphenicol 30<br>Ampicillin 2<br>Cefuroxime 30<br>Ciprofloxacin 5<br>tetracycline 30<br>Co-amoxiclav 3	28<br>18<br>27<br>30<br>25<br>50	28<br>18<br>25<br>30<br>22<br>15

Table S7. European Committee on Antimicrobial Susceptibility Testing (EUCAST) MIC cut offs for azithromycin susceptibility. MICs were measured using an agar-gradient diffusion method (the E-test) for specified bacteria

Bacteria	MIC breakpoint used (mg/L) [27]	
	Sensitive ≤	Resistant >
S pneumoniae	0.25	0.25
H. influenzae	4	4
M catarrhalis	0.25	0.25
S. aureus	2	2
